# In silico anti-alzheimer study of phytochemicals from Lamiaceae family through GSK3-β inhibition

**DOI:** 10.1038/s41598-023-47069-w

**Published:** 2024-01-08

**Authors:** Sara Zareei, Saeed Pourmand, Marzieh Eskandarzadeh, Shokoufeh Massahi

**Affiliations:** 1 Department of Cell & Molecular Biology, Faculty of Biological Sciences, Kharazmi University, Tehran, Iran; 2 Department of Chemical Engineering, Faculty of Chemical and Petroleum Engineering, University of Tabriz, PO Box: 51666-16471, Tabriz, Iran; 3 Research Committee of Faculty of Pharmacy, Lorestan University of Medical Science, Khorramabad, Iran; 4 Department of Chemistry, Faculty of Science, Ilam University, P.O. Box 69315516, Ilam, Iran

**Keywords:** Biochemistry, Computational biology and bioinformatics

## Abstract

Glycogen synthase kinase 3-beta (GSK3-β) is a serine-threonine protease expressed in the brain, and its hyperactivity is considered the underlying cause of Alzheimer’s disease. This enzyme requires an ATP molecule in its N-terminal lobe to phosphorylate its substrates, with the most important substrate being the Tau protein. This study focuses on the inhibitory mechanism of four naturally occurring compounds—apigenin, luteolin, rosmarinic acid, and salvianolic acid—from the Laminaceae family against GSK3-β. The orientation of the ligands within the ATP-binding pocket of GSK3-β and their binding energy were determined through molecular docking. Additionally, molecular dynamics simulations was conducted to study the conformational changes induced by the ligands in the protein structure. The results showed that apigenin and salvianolic acid achieved deeper parts of the cavity compared to luteolin and rosmarinic acid and formed stable complexes with the enzyme. In the rosmarinic acid complex, the enzyme exhibited the most exposed conformation. On the other hand, luteolin binding caused a small closure of the opening, suggesting a potentially ATP-competitive role. Our results suggest these compounds as lead candidates for the design of GSK3-β inhibitors.

## Introduction

Glycogen synthase kinase 3-beta (GSK3-β) belongs to the serine-threonine kinase family and is primarily expressed in the brain. It plays a regulatory role in cell signalling pathways and protein phosphorylation^1^. In the context of Alzheimer’s disease (AD), GSK3-β has drawn extensive attention due to its involvement in the phosphorylation of tau protein, a key player in the development of neurofibrillary tangles, one of the hallmark pathological features of AD^2^.

The overactivation of GSK3-β results in excessive phosphorylation of tau protein. In normal condition, tau is responsible for stabilizing microtubules within the nerve cells, aiding in the proper functioning of neural structures. However, in AD, abnormal phosphorylation of tau by GSK3-β leads to the accumulation of hyperphosphorylated tau, forming neurofibrillary tangles (NFTs) that disrupt the neural network and impair cellular process^3,4^. Structurally, GSK3-β bears a great resemblance to other protein kinases so that two distinct lobes of the N- and C- terminal are seen within its catalytic domain. The first lobe embraces ATP while the latter is important for catalysis^5^.

Lamiaceae family, also known as the mint family, holds significant importance in phytomedicine due to its beneficial effects on various conditions such as cardiovascular diseases^6^, cancer^7^, hypertension^8^, gastrointestinal disorders^9^, and diabetes^10^. This plant family exhibited anti-oxidant^11^, anti-tumor^12^, anti-fungal^11^, anti-inflammatory^13^, and pain relieving^14^ activities. Moreover, the phytochemicals derived from Lamiaceae have shown promising effects CNS-related conditions^15^. In terms of mental health, extracts from *Salvia Rosmarinus* and *Nepeta menthoides* have been found to reduce anxiety and depression^16–18^, while *Salvia officinalis* phytochemicals have shown potential in attenuating substance abuse dependency^18^. Other species, such as Plectranthus neochilus and *Salvia miltiorrhiza Bunge*, have been suggested for insomnia improvement^19^. Moreover, the sedative function of *Ballota kaiseri*, *Bystropogon maderensis Webb & Berthel.*, have been proposed previously^20,21^. Furthermore, evidence suggests that *Otostegia persica* and *Ocimum menthiifolium benth* species may provide relief for neuropsychiatric disorders like headaches, migraines, and epilepsy^22,23^. Lamiaceae members have also shown effectiveness against neurodegenerative diseases, including Parkinson’s disease, with certain species minimizing brain injury and playing a neuroprotective role^24,25^.

Among the biologically active compounds found in Lamiaceae, we have specifically chosen apigenin (AP), luteolin (LO), rosmarinic acid (RA), and salvianolic acid (SA) due to their demonstrated anti-AD effects^26^. Previous studies have also shown their ability to decrease the expression levels of GSK3-β^27–29^. Therefore, our computational study aims to investigate the potential mechanism of action of these compounds against GSK3-β, with the intention of assessing their suitability as promising lead candidates in drug design.

## Computational approaches

### Molecular docking

#### Validation

To date, the crystalized 3-D structure of GSK3-β has been identified in complex with many inhibitors. Thus, we retrieved the following PDB IDs from the RCSB data bank: 1Q41, 1Q3D, 1Q4L, 2OW3, 1R0E, 1Q3W, 3GB2, 3F88, 3F7Z, 3I4B, 1UV5, 3Q3B, 3L1S, 1Q5K, and 2O5K. The co-crystalized structures of inhibitors were removed from each ID and the protein structure was optimized using Viewer Lite 5.0. This involved removing all water molecules and non-polar hydrogens. Subsequently, each complex was subjected to a re-docking process (100 runs) using Autodock 4.2. Finally, the best ligand conformation with the lowest binding energy was selected. The RMSD of each ligand between its crystalized and re-docked conformations were calculated by Visual Molecular Dynamics (VMD) 1.9.3 software. Finally, the PDB ID with the minimum RMSD was selected for GSK 3-β-ligand docking based on the validation results.

#### Ligand preparation

The 3D structures of apigenin, luteolin, rosmarinic acid, and salvianolic acid were obtained from the PubChem database and underwent an energy minimization step using the MOPAC program in ChemDraw Ultra 8.0. In the next step, the Gasteiger charge was added, while nonpolar hydrogens were removed. Additionally, rotatable bonds were defined and considered flexible.

#### Protein preparation

The A chain of protein structure from the selected PDB from the validation step with the lowest RMSD was extracted by removing all water molecules and co-crystallized ligands using the VierwerLite 5.0 tool. In the end, the protein’s Kollman charge was determined.

#### Docking procedure

The ligands and the target protein were docked using the Lamarkian genetic algorithm (LGA) and the Auotodock 4.2. program with 100 runs. The x, y, and z coordinates of the grid box center for all ligands were set as 94.63, 68.168, and 9.788, respectively. The sizes of the grid boxes were defined as 56 × 64 × 54 for apigenin, 50 × 54 × 44 for luteolin, 66 × 68 × 52 for rosmarinic acid, and 54 × 62 × 46 for salvianolic acid.

Regarding ATP, we used PDB ID 1PYX, in which the enzyme is bound to AMP-PNP, a stable form of ATP with the gamma phosphor atom substituted by nitrogen. The coordinates of this molecule were used to determine the dimensions of the grid box grid box as 63 × 44 × 39 and the grid center as 89.237, 67.513, and 9.829 in x, y, and z directions, respectively. In other words, the docking was performed at the exact location on the enzyme as demonstrated in the crystalized structures but using the identical protein structure as the other dockings.

#### Molecular dynamics simulation

The simulation was carried out using GROMACS 2019.1 with Ubuntu 18.04 Linux, running on an Intel core 12 Quad 6800k 3.6GhHZ, GPu: Nvidia GeForce GTX 1080Ti, and with 16GB RAM. The apo-GSK3-β and ligand-bound GSK3-β with the lowest binding energy were simulated, and three independent repeats of the simulations were performed for 200 ns. The topology and coordinate files were generated using the Charmm27 force field by gmx pdb2gmx and the SwissParam server for the protein and ligands, respectively. In detail, each system was placed in a cubic box filled with extended simple point-charge (SPC) water molecules under periodic boundary conditions, with a 1 nm distance from each edge. Electrostatic neutrality of the systems was achieved by adding chloride and sodium ions.

In the subsequent stage, energy minimization was carried out in 50,000 steps for 2 fs. Then, the system was equilibrated at a stable temperature of 300 K using the Brendsen temperature algorithm and pressure of 1 bar in the NVT NPT ensembles. Finally, the 200 ns MD production was carried out using the mesh Edward (PME) method and Verlet algorithm to define long-range electrostatic forces and calculate trajectories, respectively. Root-mean-square deviation (RMSD), root mean square fluctuation (RMSF), radius of gyration (Rg), solvent-accessible surface area (SASA), hydrogen bond analysis, Molecular mechanics Poisson–Boltzmann surface area (MM-PBSA) were calculated to analyze the trajectories. Additionally, Principal Component Analysis (PCA) was also calculated using gmx covar and gmx anaeig to create a covariance matrix and diagonalizing it, and generate projections onto principal components. All parameters and analysis presented in the results are the average values of replicates.

## Results and discussion

**Figure 1:**
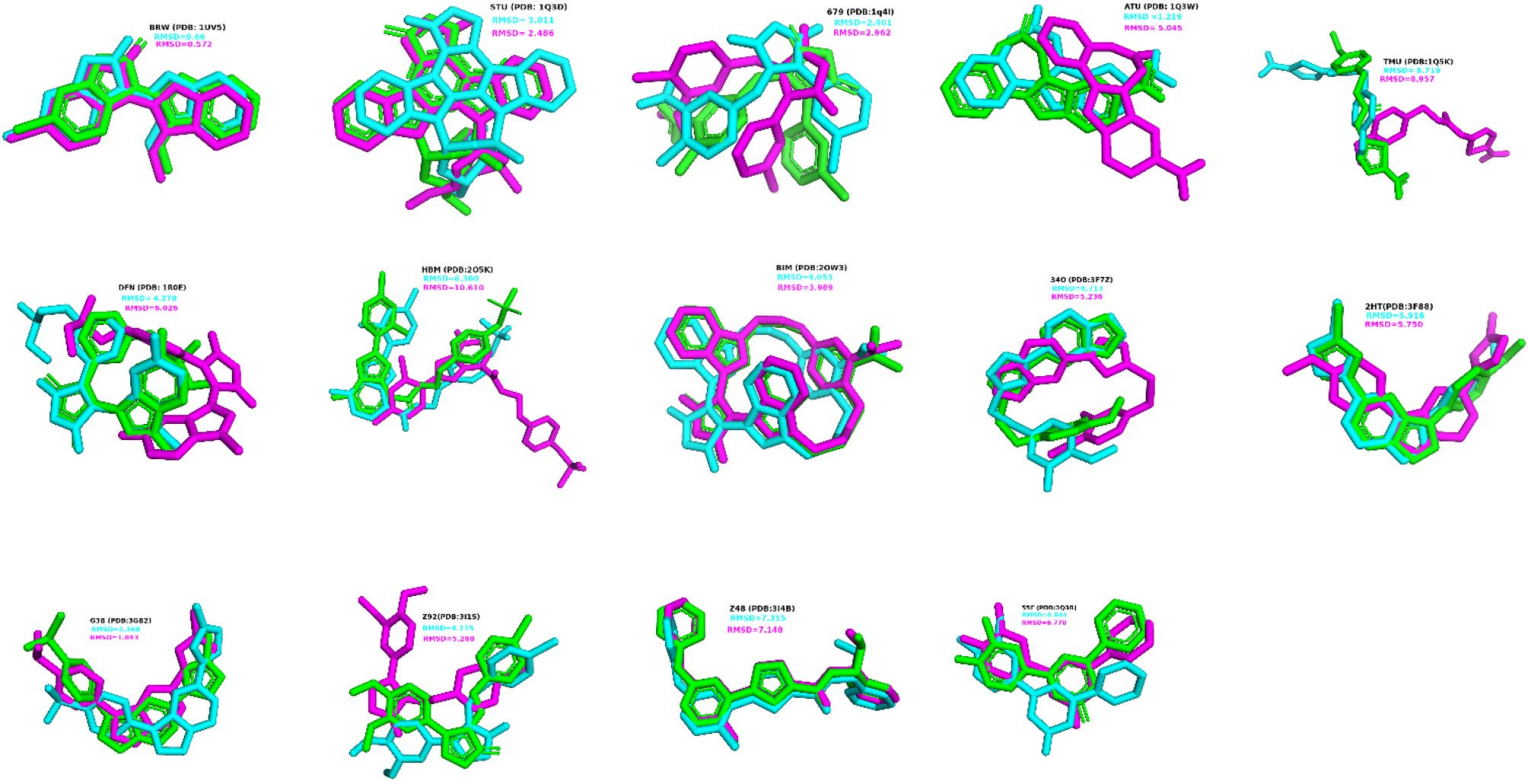
Docking validation- RMSD and poses of crystallized (green), AutoDock- (cyan), and AutoDock Vina- (magenta) generated structures.

**Figure 2:**
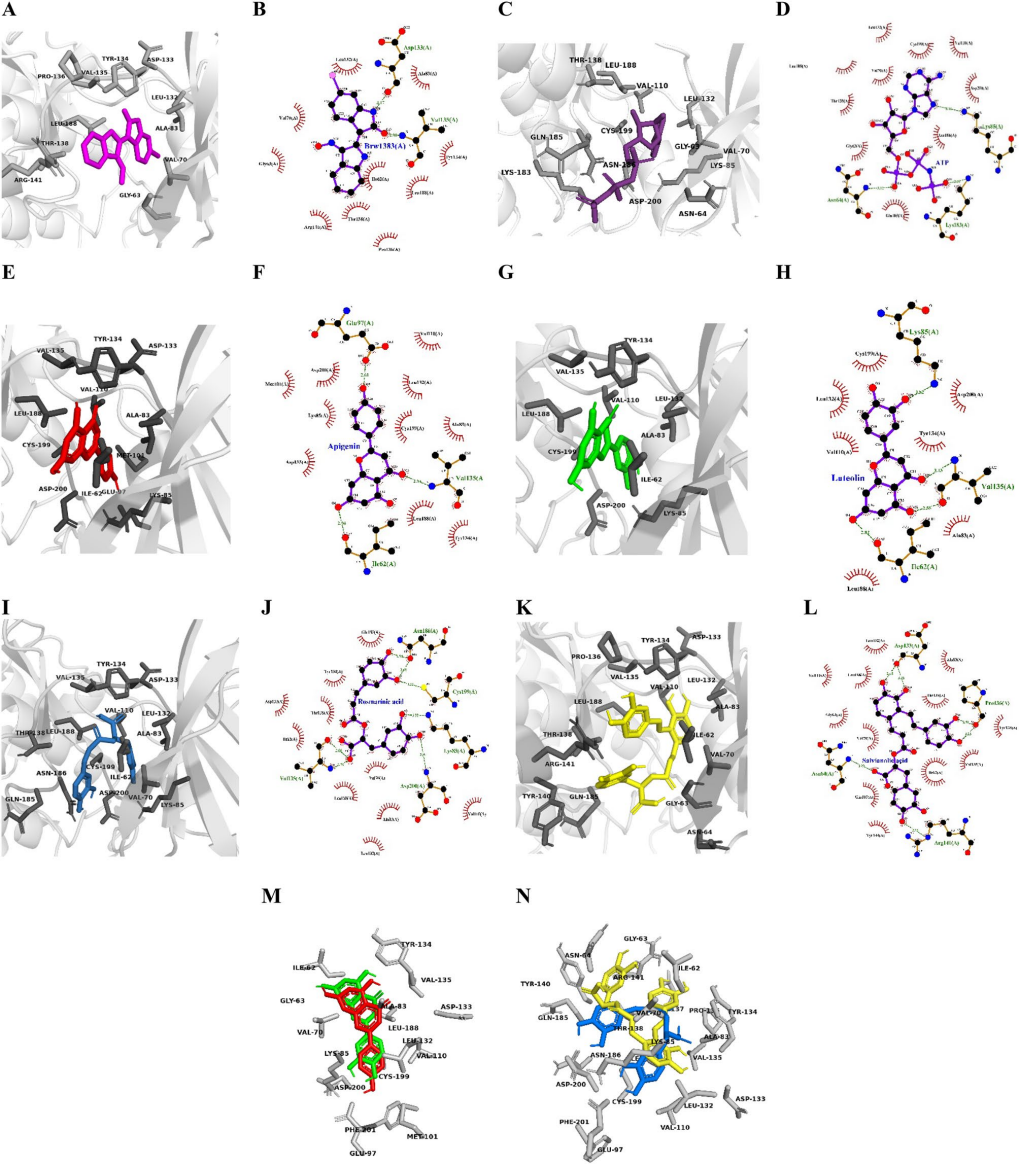
3D and 2D binding interactions of Brw1383 (**A** and **B**), ATP (**C** and **D**), apigenin (**E** and **F**), apigenin (**G** and **H**), rosmarinic acid (**I** and **J**), and salvianolic acid (**K** and **L**). The ligands are represented as magenta, violet, red, green, blue, and yellow, respectively while the receptor is depicted in the gray transparent cartoon. Superimpositions of apigenin and luteolin (**M**) as well as rosmarinic acid (blue) and salvianolic acid (yellow) and GSK3-β residues (white) (**N**) surrounding 4 Å.

The present study is devoted to the computational evaluation of the inhibitory mechanism of action of four compounds occurring in the Lamiaceae family against the excessive activity of GSK3-β, which is associated in Alzheimer’s disease. The docking protocol was validated by docking approved inhibitors against the enzyme using Autodock and Autodock Vina programs. Generally, docking simulations yielding a RMSD of less than 1.5 or 2 Å when comparing computational and known ligand poses are considered effective implementations^30^. The present results showed that docked pose of PDB ID 1UV5, with the binding energy of − 9.56kcal/mol, had the lowest RMSD (0.66) in comparison with others (Fig. 1). In this structure, the ATP-binding region of GSK3-β is inhibited by 6-Bromione dirubio-3-oxime (BRW1383) which made connections with receptor’s Ile62, Gly63, Val70, Ala83, Leu132, Asp133, Tyr134, Val135, Pro136, Thr138, Arg141, and Leu188 (Fig. 2A and B). All bonds were identical to the docked pose, suggesting the reliability of the prediction of ligand binding sites made by docking protocol.

Before examining the ligands, we performed a docking simulation of the ATP molecule within the ATP-binding cavity to assess the potency of the enzyme's natural substrate. The result showed that ATP binds Gly63, Asn64, Val70, Lys85, Val110, Leu132, Thr138, lys183, Glu185, Asn186, Leu188, Cys199, and Asp200 (as illustrated in Fig. 2C and D). Additionally, the binding energy of this interaction was determined to be − 6.92 kcal/mol. The docking results are consistent with previously reported residues Ile62, Val70, Ala83, Val133, Tyr134, on one side, and Thr138, Arg141, Leu188, Cys199^5^.

The AP, LO, RA, and SA ligands were docked against the ATP-binding pocket of GSK3-β, and their best binding conformations yielded binding energies of − 8.28, − 8.08, − 7.50, and − 8.98 kcal/mol, respectively. This suggests that all the ligands can compete with ATP, the natural substrate of the enzyme, and therefore they could be considered potent competitive inhibitors. Among the compounds, SA exhibited the most negative binding energy, indicating stronger binding affinity, while RA had the most positive energy. This implies that while the first ligand had a lower energy barrier to locate the ATP-binding, the latter ligand needs to overcome a higher energy barrier to inhibit GSK3-β. The enhanced potency of SA might be attributed to its higher number of hydrogen bonds formed with Asn64, Asp133, Pro136, and Arg141 compared with the other ligands (Fig. 2K and L).

The docking results of the compounds are shown in Fig. 2A–N. It was observed that all ligands interacted with the same set of residues with minimal variation. AP formed three hydrogen bonds with receptor’s Ile62, Glu97, and Val135, involving its O_3_, O_4_, and O_5_ atoms (Fig. 2E and F). This interaction pattern is comparable to luteolin, which has an additional OH substitution bound with C21. However, this functional group did not establish a hydrogen bond with the surrounding residues. Instead, a new hydrogen bond was formed between O_2_ and Val135. It can be noticed that a higher number of hydrogen bonds could not guarantee higher potency in LO (Fig. 2G and H). Comparing the residues surrounding AP and LO, it can be perceived that AP achieved deeper parts of the ATP binding cavity. This may suggest that bulky substitutions in the B ring might have difficulty reaching deep into the channel and potentially contribute to the increase in the binding energy. Conversely, residues located distally from the surface effectively retain the ligand through a higher number of hydrophobic interactions. This is evident in AP with 10 interactions involving Ala83, Lys85, Met101, Val110, Leu132, Asp133, Tyr134, Leu188, Cys199, and Asp200, compared with LO with 7 interactions involving Ala83, Val110, Leu132, Tyr134, Leu188, Cys199, and Asp200 (Fig. 2F and H).

Previous studies have demonstrated the anti-Alzheimer's disease (AD) activities of AP through various mechanisms. For instance, AP has been shown to reduce insoluble Aβ levels and improve memory and learning deficits in AD mouse models by modulating β-C-terminal fragment (β-CTF) and β-site AβPP-cleaving enzyme 1 (BACE1)^31^. AP treatment in AD mice has also been observed to inhibit the production of LPS-induced IL-6 and TNF-α, indicating its potential in modulating neuroinflammation associated with AD. Docking studies suggest that GSK3-β’s inhibition may be another molecular mechanism underlying these experimental results, given the role of GSK3-β in these pathways. It is worthy of note that AP exhibits favorable permeability^32^ and can cross the blood–brain barrier in its potassium salt form^33^. Although this makes it a druggable candidate for brain delivery, several carriers such as ethosomes and carbon nanopowder have been proposed to enhance the efficacy of AP^34,35^.

Both RA and SA also have the potential for GSK3-β suppression, as these ligands showed to occupy the ATP binding pocket, with SA exhibiting a more negative binding energy. It can be noticed from Fig. 2I–L that these ligands followed discrepant hydrogen bonding patterns. RA forms seven h-bonds with Lys85, Val135, Asn186, Cys199, and Asp200, while SA establishes hydrogen bonds involving Asn64, Asp133, Pro136, and Arg141 for constructing six hydrogen bonds.

Rosmarinic acid, formally known as (R)-α-[[3-(3,4-dihydroxyphenyl)-1-oxo-2 E-propenyl]oxy]-3,4-dihydroxy-enzenepropanoic acid, has remarkable beneficial effects against various conditions^36^, including cancer, diabetes, hepatotoxicity, and depression^37–40^. Its effects on memory and learning have been investigated in several studies using AD models, which demonstrated that RA treatment improved these cognitive functions^41^. In middle-aged mice, RA proved to reduce the phosphorylation levels of tau protein, a reaction catalyzed by GSK3-β that leads to the insolubility of tau^42^. Our docking results imply that GSK3-β’s inhibition may be the underlying mechanism for such experimental outcomes.

Salvianolic acids (SA) naturally occur in ten different classes, ranging from A to J. Some classes consist of fusions of Danshensu and caffeic acid, while others contain only caffeic acid. Generally, salvianolic acids exhibit well-established bioactivities against fibrosis, cancer, cardiovascular diseases, and AD^43–45^. In this study, we specifically evaluated the inhibitory potential of SA type A (SAA) which is composed of a Danshensu and two caffeic acids^46^. In the context of CNS diseases, SAA treatment has been shown to decreases inflammation and apoptosis, promotes neurogenesis, and protect the blood–brain barrier in the ischemic brain^47,48^. SAA has also demonstrated beneficial effects in the treatment AD, inhibiting Aβ42 aggregation and disaggregating pre-formed fibrils^49^.

Referring to the docking results, it can be inferred that SAA benefited significantly from its three dihydroxy phenyl moieties, which allowed it to anchor to both deeper and surface residues. As shown in Fig. 2K, Danshensu’s ring positioned itself at the bottom, while the ring of caffeic acids interacted with the opening residues, inlcuding Ile62, Ala 83, Tyr134, Arg141, and Leu188. However, RA consisted of only two rings, one dihydroxy phenyl on one side and another from dihydroxy benzene propanoic acid on the other side. Despite establishing higher numbers of hydrogen bonds with receptors, RA reached the bottom of the channel without surpassing SAA (Fig. 2M). This indicates that the comprehensive network of connections predominantly established through hydrophobic interactions might offer an advantage over hydrogen bonding. Similarly, AP and LO had a significant number of hydrogen bonds, although their binding energy was lower than that of SA’s.

Paudel et al., demonstrated the inhibitory effect of LO, SA, and RA on the enzyme, suggesting that SA is more potent than RA, which aligns with our findings. However, their results indicating that LO is more potent than SA and RA contradict our results. This inconsistency calls for further investigation^50^. In a computational study, the results obtained from induced-fit molecular docking of RA, AP, and SA were similar to ours. Both the docking and ΔG_binding_ analyses indicated that SA was more potent than AP and RA, with RA displaying the lowest potency^51^. Elekofehinti et al., also investigated the potential of LO and RA on the GSK 3-beta active site. Although their docking results suggested higher potency of LO compared to RA, contradicting our findings, their ΔG_binding_ calculations yielded opposite results^52^. None of these computational studies provided a mechanism of action for their ligands.

### MD simulation

In addition to providing detailed information about ligand–protein interactions using molecular docking, we sought to decipher the changes that occur in protein conformation following the formation of ligand–protein complexes. Molecular dynamics (MD) simulations can shed light on the dynamics and behavior of atoms within a system^53^. In the present study, all systems underwent three rounds of 200 ns of simulations.

**Figure 3:**
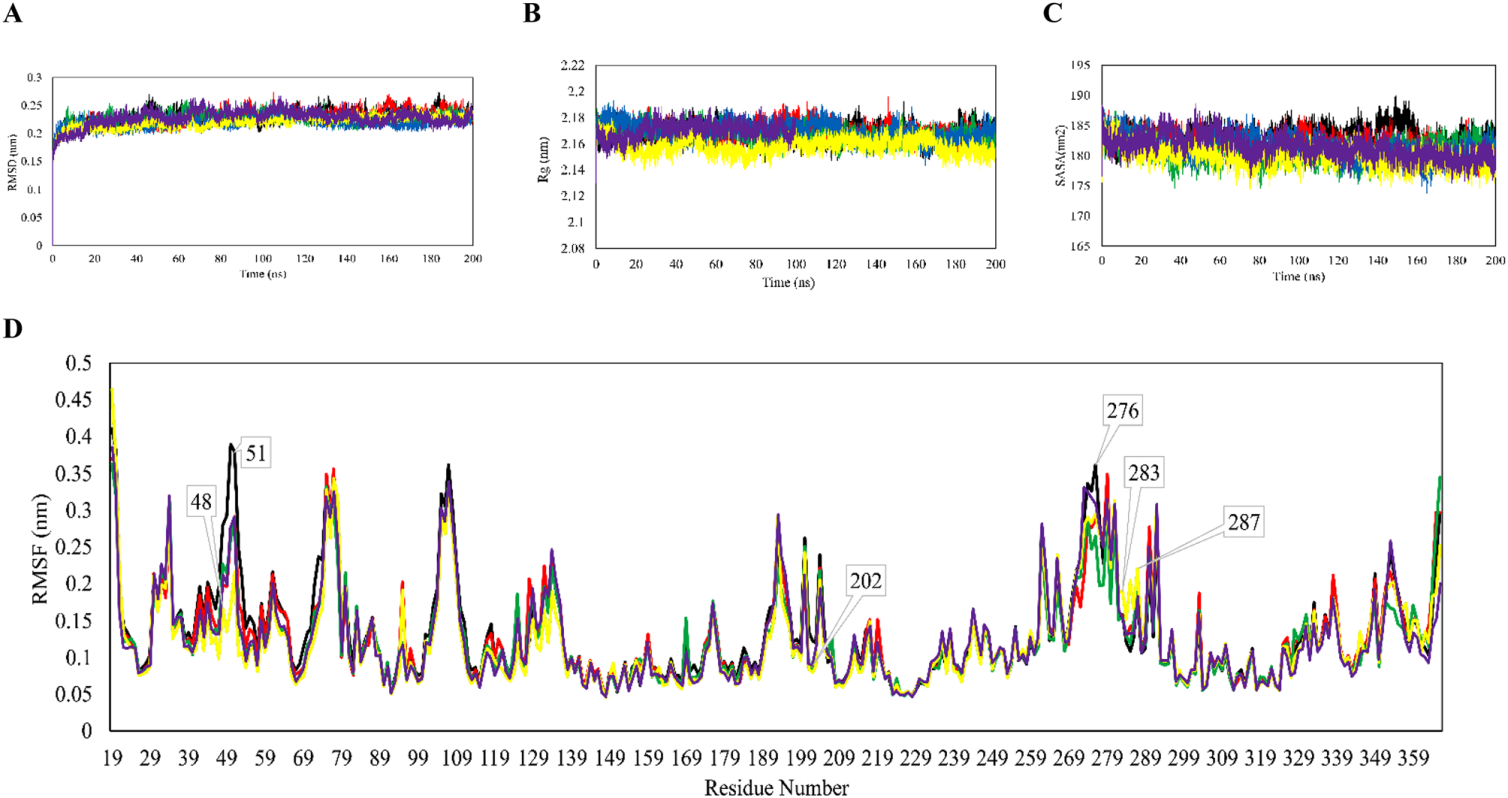
MD analysis of GSK3-β in apo (black) and in complex with ATP (violet), AP (red), LO (green), RA (blue), and SA (yellow). Average (**A**) RMSD, (**B**) Rg, (**C**) SASA, and (**D**) RMSF analyses of triplicates.

Initially, the stability of an inhibitor-enzyme complex holds significant importance in determining the extent of inhibition. The greater the stability of an inhibitor in forming a complex with the enzyme, the more likely it is to induce irreversible inhibition. RMSD, a parameter indicating stability and structural changes during MD, quantifies the differences in atom position between initial and final frames. Higher deviations or fluctuations observed in RMSD plots reflect significant conformational changes through the simulation, indicative of molecule instability. RSMD plots of GSK3-β Cα were calculated after extracting MD trajectories of three replicates and the average values were plotted (Fig. 3A). All systems reached equilibrium within 10 ns, except for RA and ATP, which displayed notable fluctuations during the simulation. These unstable plots suggest that the ligand binding induces significant changes in the enzyme conformation compared to the apo- and other ligands-bound receptor, although no denaturation or deformation is observed. A comparison between ATP and RA reveals that the former molecule exhibits relatively greater stability, suggesting that the enzyme undergoes significant conformational changes upon binding to its substrate. Furthermore, the results suggest that the ligands AP, LO, and SA not only block the active site but also exert an additional inhibitory effect by restricting protein motion. RA forms the most stable complex with the enzyme even surpassing the stability of the apo-enzyme. The mean average RMSD values for GSK3-β and its complexes with ATP, AP, LO, RA, and SA were 0.228, 0.227, 0.229, 0.226, 0.219, and 0.220, respectively.

After examining protein stability, we next sought to gain deeper understanding of the impact of these small ligands have on inducing conformational changes in GSK3-β through Rg and SASA calculations, both of which indicate protein dimension and compactness. As shown in Fig. 3B and C, all ligands influenced protein conformation, resulting in increased values of Rg and SASA upon binding of AP, LO, RA, SA, and ATP. Notably, when bound to RA and ATP, GSK3-β exhibited the most exposed conformation, as consistent results from RG and SASA suggest.

To identify the specific residues contributing to conformational deviation and instability upon ligand binding, we performed RMSF, as shown in Fig. 3D. Remarkably, active site residues demonstrated minimal fluctuations, indicating their low mobility. Notably, the catalytic loop of GSK3-β (residues 180-214)^54^ remained relatively similar across all systems, except for residue 202, which displayed decreased volatility upon ligand binding, suggesting reduced mobility during the 200 ns simulation.

**Figure 4:**
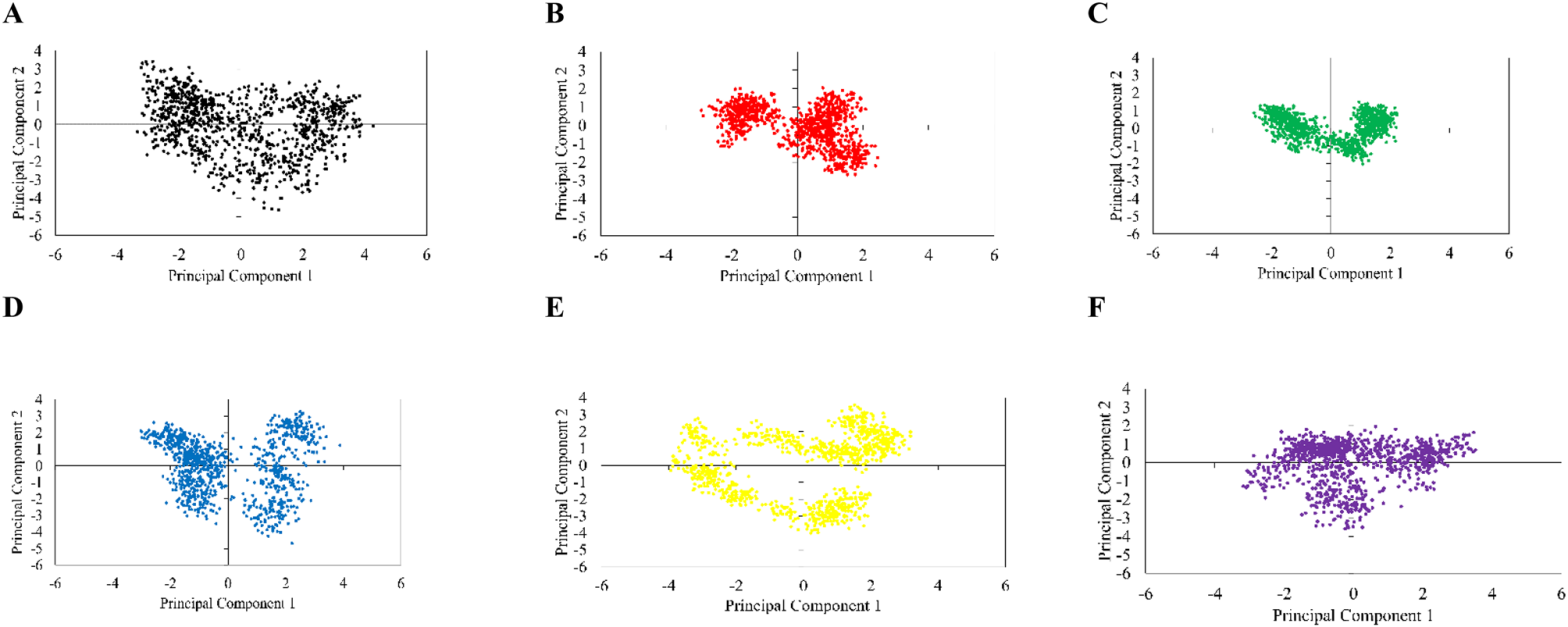
Projection Plots of average Principal Component Analysis of triplicates of GSK3-β (**A**), apigenin (AP) (**B**), luteolin (LO) (**C**), rosmarinic acid (RA) (**D**), salvianolic acid (SA) (**E**), and ATP (**F**): exploring conformational space and dynamics during the 200-ns simulaitons.

Further evaluation of conformational changes in GSK3-β was carried out through PCA analysis, as it effectively reduces the high dimensionality of huge related data such as atom coordinates and dihedral angles over the simulation^55^. The trajectory projection plot shows that the poins’ positions in the plot are centered around the origin (Fig. 4), indicating that all systems undergo fluctuations around an equilibrium state during the simulations. Moreover, all ligands resulted in more compact PCA plots for GSK3-β, whereas apo-protein has the most expanded PCA plot among all the ligand-bound forms. This observation suggests that in the absence of any ligand, the protein displays inherent flexibility and can explore a wider range of conformational states. Conversely, the presence of ligands causes the protein conformations to become more compact and clustered together, as supported by Rg and SASA plots. As it is seen in Fig. 4E, RA has a more expanded PCA plot compared to the other ligands. This indicates that the binding of RA induces larger conformational changes in the protein structure.

Further exploration of conformational change can be achieved by using a contact map, which provides a perspective of residues that are spatially close but sequentially distant. This proximity is known to play a crucial role in protein folding^56^. The resulting distance maps showed no significant change in distance patterns between apo- and ligand-bound states of GSK3-β, indicating that ligands induce localized conformational change in the enzyme (Fig. S1).

However, certain inhibitors have been reported to induce a conformational shift in GSK3-β. For instance, the pS9 peptide induces an approximate shift of 8.5 Å in the C-loop (residues 91-96), which corresponds to the opening domain of the ATP-binding cavity, moving it towards the C-terminal lobe^57^. Considering the present results, it is evident that conformational changes occur during the simulation period. To assess whether these movements can affect ligands bonding, changes in active site residues that were involved in ligands’ binding is shown in Table 1. The results suggest that SA forms the most stable bonds with active site residues, while RA experiences a loss of most interactions with the enzyme’s active site. Moreover, none of the residues tend to bind with the substrate binding site, namely Arg 96, Lys 205, and Arg 189^58,59^. This evidence suggests that these ligands may be rather ATP-competitive inhibitors of GSK3-β.

**Table 1:** Changes in bond formation between ligands and GSK3-β residues after 200 ns of MD simulations.

Ligands complexed with GSK3-β	Newly connected residues	Stable residues	Late residues
Apigenin	Val70, Thr138	Ala83, Lys85, Leu132, Tyr134, Val135, Cys199, Asp200	Ile62, Glu97, Met101, Val110, Asp133, Leu188
Luteolin	Gly65, Val70, Asp133, Pro136	Ile62*, Leu132, Tyr134, Val135, Leu188, Cys199, Asp200**	Ala83, Lys85, Val110,
Rosmarinic acid	Val72, Arg141	Ile62, Ala83, Leu132, Asp133**, Tyr143**, Val135^¥^, Leu188	Val70, Lys85, Val110, Thr138, Gln185, Asn186, Cys199, Asp200
Salvianolic acid	Gly137, Cys199	Ile62, Val70, Ala83, Val110, Leu132, Asp133^¥^, Tyr134, Val135**, Pro136^¥^, Thr138, Tyr140, Arg141*, Gln185, Leu188	Gly63, Asn64,
ATP	Ile62, Gly65, Phe67, Gly68, Glu97, Met101, Asp133, Phe201	Gly63, Asn64, Val70, Lys85, Leu132, Asp200	Thr138, Lys183, Glu185, Asn186, Leu188, Lys199

The number of hydrogen bonds is determined by either single or double underlines. ¥ signifies the loss of one of the late double H-bonds while * is used to determine the loss of a single H-bond. ** stands for newly formed hydrogen bonds.

**Figure 5:**
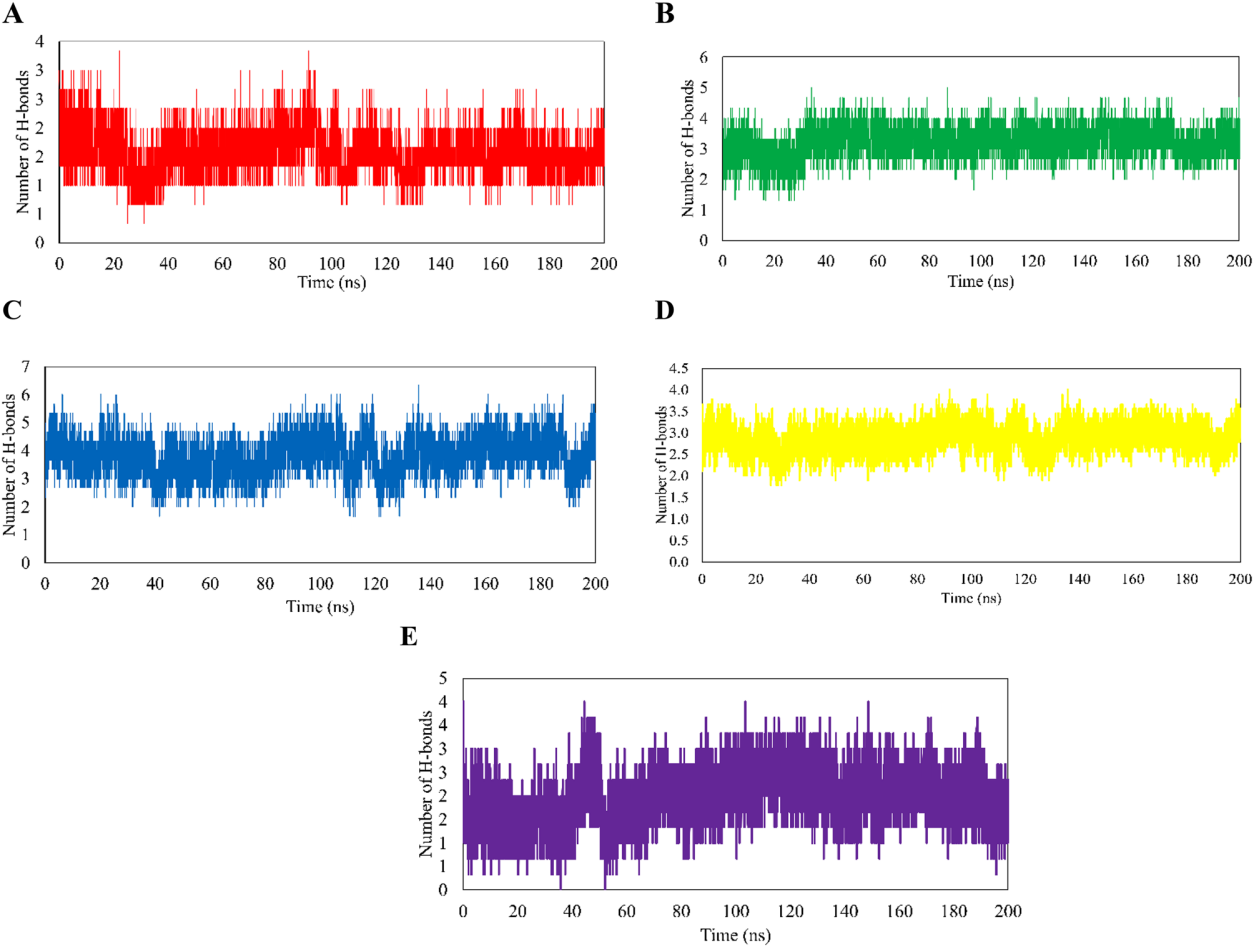
Average HBond Analysis of apigenin (AP, **A**), luteolin (LO, **B**), rosmarinic acid (RA, **C**), and salvianolic acid (SA, **D**)-GSK3-β in terms of the number of hydrogen bonds. The frequency of hydrogen bonds is plotted as the average number of bonds in three replicas of each system.

Notably, the directional and strength of the hydrogen bonding play a pivotal role in the formation and stabilization of inhibitor-enzyme complexes. Table 1 shows the lost and newly constructed H-bonds before and after MD simulations. Moreover, Fig. 5 shows the alterations in the average number of H-bonds across replicas, serving as an indicator of the stability of these interactions. Interestingly, despite SA having the lowest docking energy, its hydrogen bonds were less stable (Fig. 5D) compared to the other ligands (Fig. 5A–C, and E), highliting the significant role of hydrophobic interactions in the enzyme inhibition by SA.

Lastly, in order to determine the most potent ligand, we employed MMPBSA (Molecular Mechanics Poisson Boltzmann Surface Area) (Table 2), a method used to calculate free energies of binding for protein–ligand complexes. This table presents the average MMPBSA values of AP, LO, RA, SA, and ATP, and their binding energies with GSK3-β. The binding energies are measured in terms of van der Waals energy, electrostatic energy, polar solvation energy, SASA energy, and the total binding energy, which includes the standard deviation of values of three replicates.

**Table 2:** Average MMPBSA values of apigenin (AP), luteolin (LO), rosmarinic acid (RA), and salvianolic acid (SA)-GSK3-β binding energies including van der Waals, electrostatic, polar solvation, SASA, and total binding energy ± standard deviation of three replicates.

Energy	AP	LO	RA	SA	ATP
Van der Waal energy	− 124.497 ± 2.51	− 124.5 ± 2.51	− 116.346 ± 6.99	− 206.390 ± 6.691	− 194.945 ± 40.81
Electrostatic energy	− 83.033 ± 6.48	− 83.033 ± 6.48	− 125.305 ± 27.41	− 54.650 ± 3.614	− 149.646 ± 26.48
Polar solvation energy	93.456 ± 82.18	140.564 ± 1.26	199.055 ± 26.88	187.020 ± 14.419	305.146 ± 62.79
SASA energy	− 9.325 ± 6.08	− 16.066 ± 6.16	− 12.906 ± 5.67	− 12.485 ± 8.482	− 19.162 ± 2.18
Total binding energy	− 77.982 ± 8.82	− 83.032 ± 1.56	− 55.503 ± 4.12	− 86.503 ± 1.432	− 58.606 ± 4.30

Apigenin, luteolin, and ATP have similar van der Waals energies, while rosmarinic acid and salvianolic acid have higher energies. This suggests the presence of attractive or repulsive forces between atoms that are not involved in covalent bonds, contributing to the binding of these ligands.

Apigenin demonstrated the lowest polar solvation energy, while luteolin, rosmarinic acid, and salvianolic acid had higher energies. ATP has the highest polar solvation energy, indicating a greater degree of solvation. The total binding energy supports the docking analysis and MD simulations since it is observed that SA had the most negative energy of binding.

## Conclusion

Among the naturally occurring compounds apigenin, luteolin, rosmarinic acid, and salvianolic acid from the Laminaceae family, SA, and LO showed the highest affinity for the ATP-binding site of GSK3-β. AP and SA were able to achieved deeper regions of the cavity compared to LO and RA. Moreover, these two ligands formed stable complexes with the enzyme, although all ligands induced conformational changes in the protein. Notably, the RA complex exhibited the most exposed conformation.

Based on PCA analysis, GSK3-β exhibits inherent flexibility in the absence of any ligands, allowing it to explore a wider range of conformational states. However, in the presence of ligands, the protein conformations became more compact and clustered together. Moreover, the binding of RA induced larger conformational changes in the protein structure, highlighting its biological significance as a ligand for the protein.

### Supplementary Information

Supplementary Figures.

## Data Availability

The protein structures analyzed during the current study are available in the RCSB data bank with PDB IDs: 1Q41, 1Q3D, 1Q4L, 2OW3, 1R0E, 1Q3W, 3GB2, 3F88, 3F7Z, 3I4B, 1UV5, 3Q3B, 3L1S, 1Q5K, and 2O5K. In order to ensure transparency and reproducibility, we have uploaded the primary data onto GitHub (https://github.com/SaraZareei/anti-Alzheimer-s-ligands.git). The deposited data includes the PDB files for Figs. 1 and 2, the XVG files for Figs. 3, 4, and 5, as well as the MDP files for the production simulations and the control parameter file for the docking study. Additionally, we have added the starting complexes of the simulations for readers’ reference.

## Abbreviations

GSK3-β: Glycogen synthase kinase3 beta
ATP: Adenosine triphosphate
AP: Apigenin
LO: Luteolin
RA: Rosmarinic acid
SA: Salvianolic acid
AD: Alzheimer’s disease
CNS: Central nervous system
MD: Molecular Dynamics
RMSD: Root-mean-square deviation
Rg: Radius of gyration
SASA: Solvent-accessible surface area
RMSF: Root mean square fluctuation
PCA: Principal Component Analysis
MM-PBSA: Molecular mechanics Poisson–Boltzmann surface area
LGA: Lamarkian genetic algorithm
SPC: Simple point-charge
NVT: Constant-temperature, constant-volume ensemble
NPT: Constant-temperature, constant-pressure ensemble
LPS: Lipopolysaccharide
TNF-α: Tumour necrosis factor α
IL-6: Interleukin-6
