# Supplementary material for: In silico anti-alzheimer study of phytochemicals from Lamiaceae family through GSK3-β inhibition
Source: Sci Rep. 2024 Jan 8;14:834. doi: 10.1038/s41598-023-47069-w (PMC10774376; doi:10.1038/s41598-023-47069-w)
Supplement: Supplementary file 1 — Supplementary Figures. [file 41598_2023_47069_MOESM1_ESM.docx]

| A |  |  |
| --- | --- | --- |
| Replica1 | Replica2 | Replica3 |
| 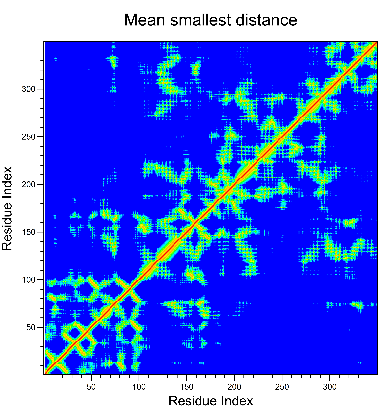 | 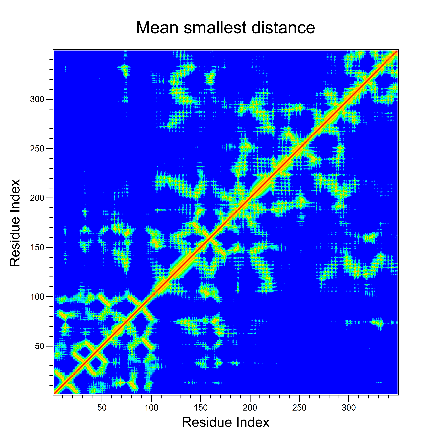 | 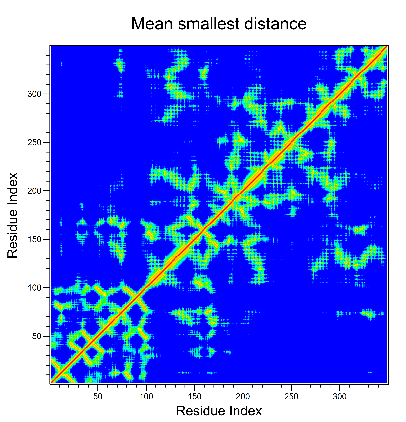 |
| B |  |  |
| 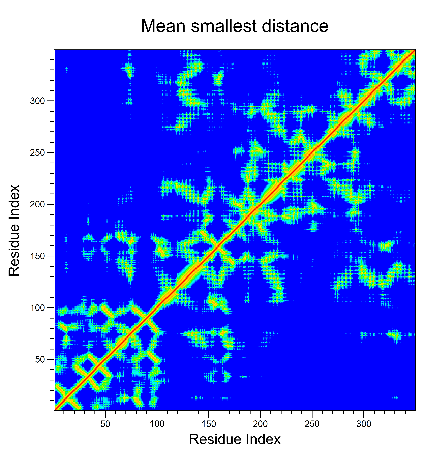 | 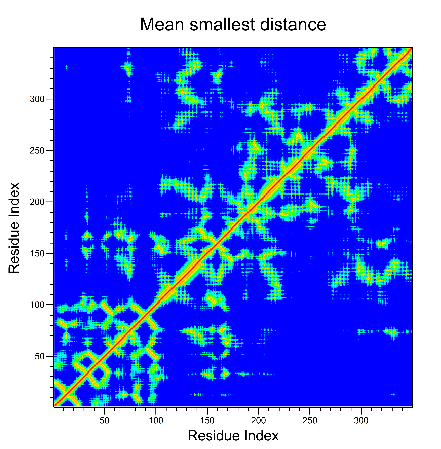 | 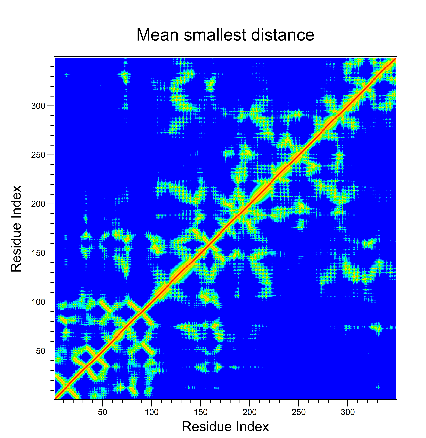 |
|  |  |  |
| C |  |  |
| 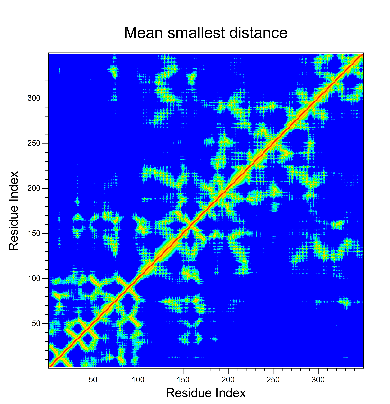 | 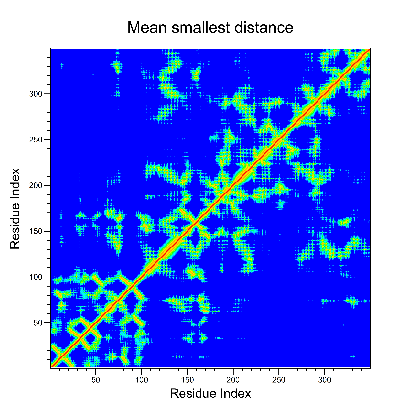 | 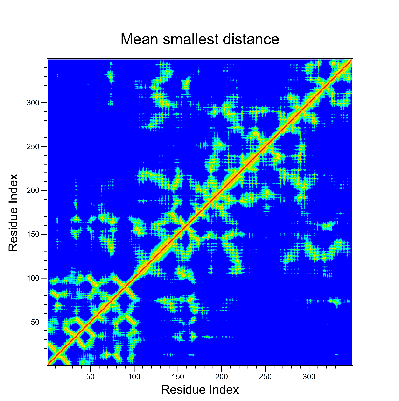 |
| D |  |  |
| 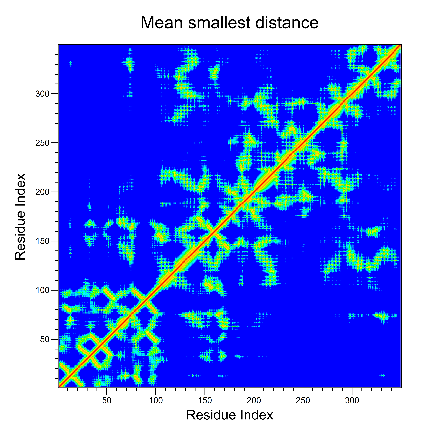 | 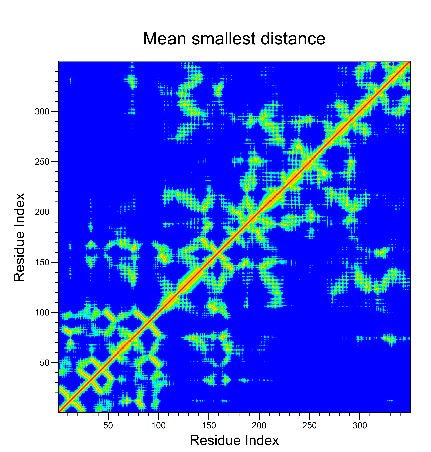 | 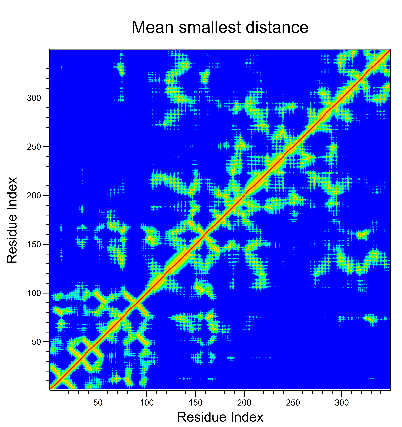 |
| E |  |  |
| 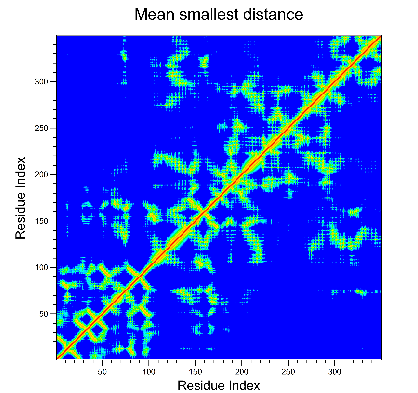 | 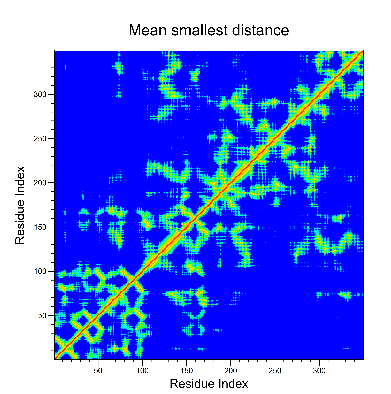 | 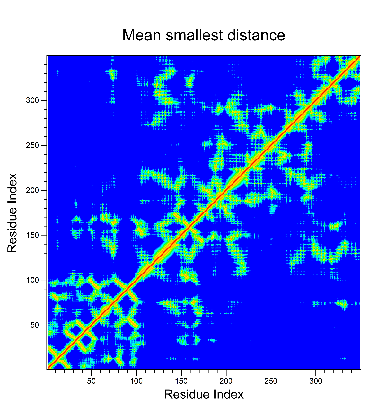 |
| F |  |  |
| 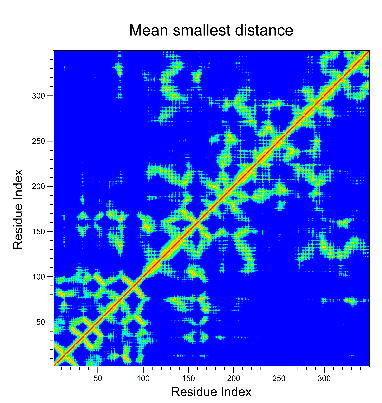 | 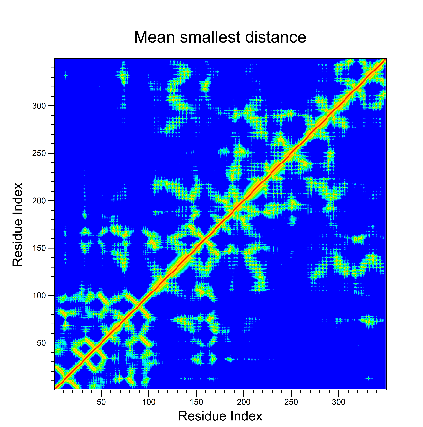 | 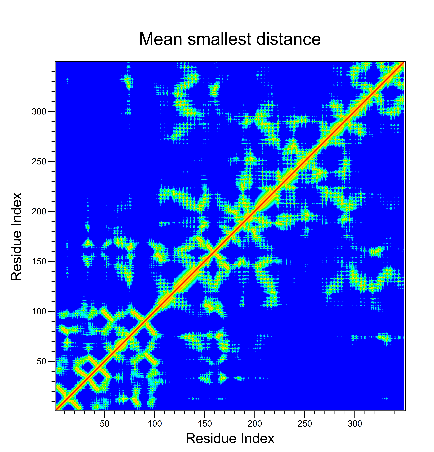 |
|  |  |  |
| Fig. S1- Contact Map Analysis of three replicates (trajectories 1-3) of GSK3-β (A), ATP (B), apigenin (AP) (C), luteolin (LO) (D), rosmarinic acid (RA) (E), and salvianolic acid (SA) (F): Identification of Closest Residue Contacts after 200-ns simulaltion. | | |

| A |  |  |
| --- | --- | --- |
| Replica1 | Replica2 | Replica3 |
| 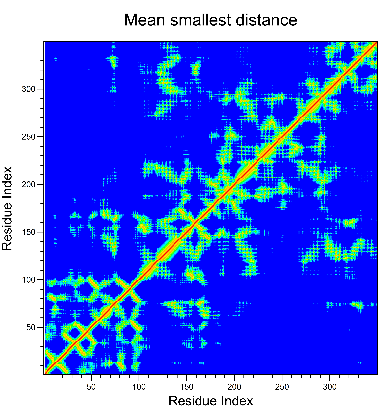 | 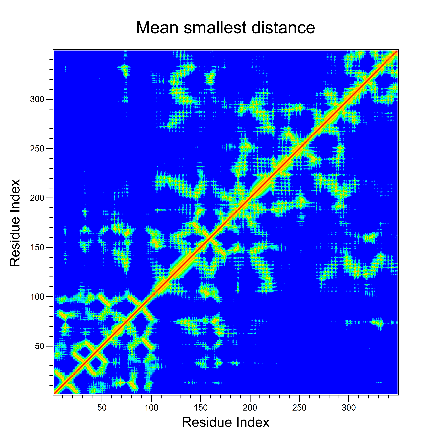 | 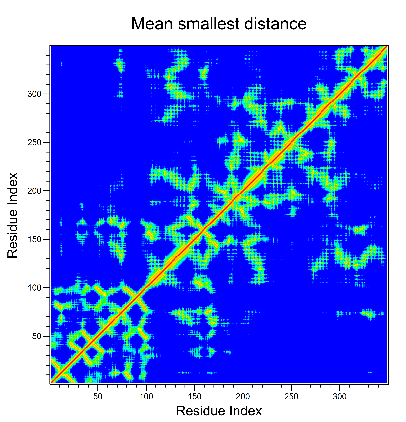 |
| B |  |  |
| 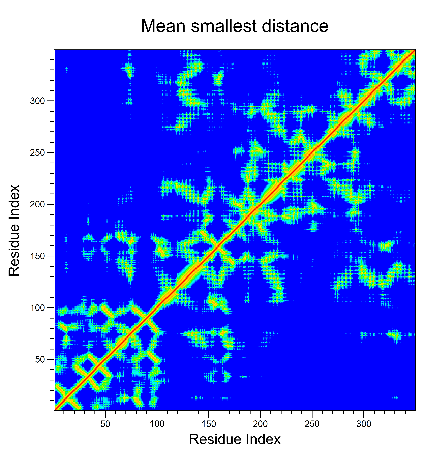 | 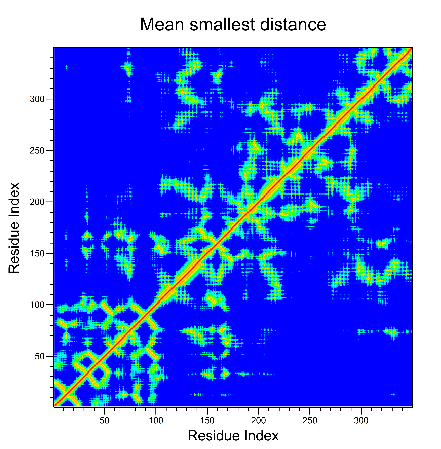 | 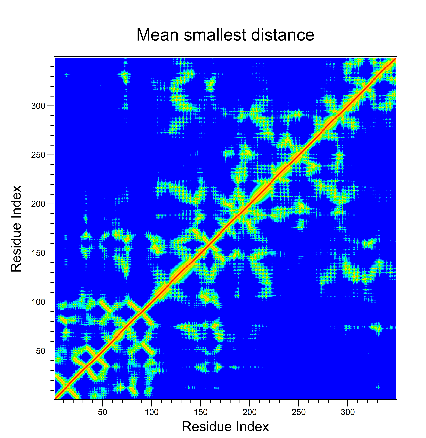 |
|  |  |  |
| C |  |  |
| 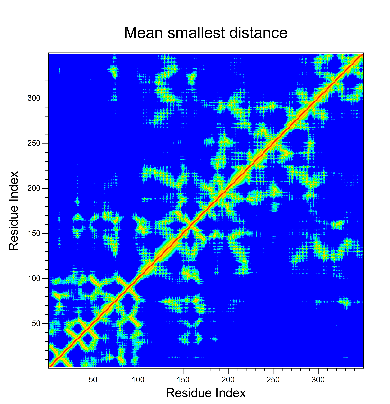 | 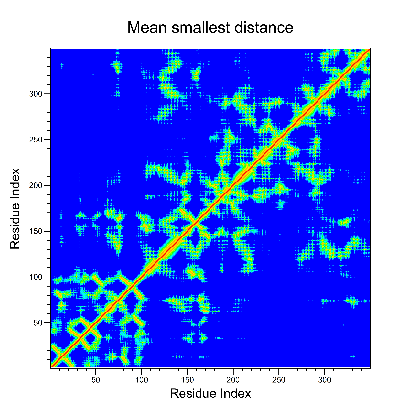 | 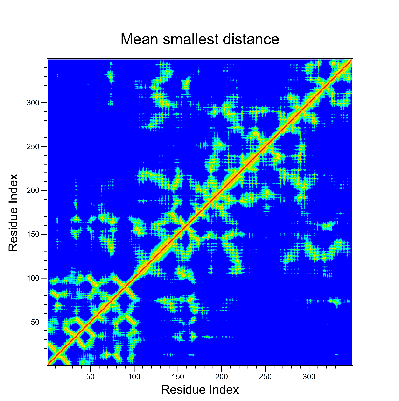 |
| D |  |  |
| 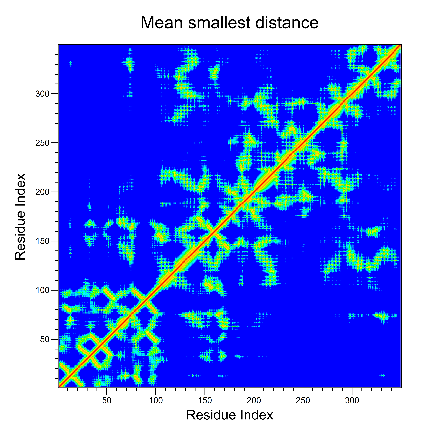 | 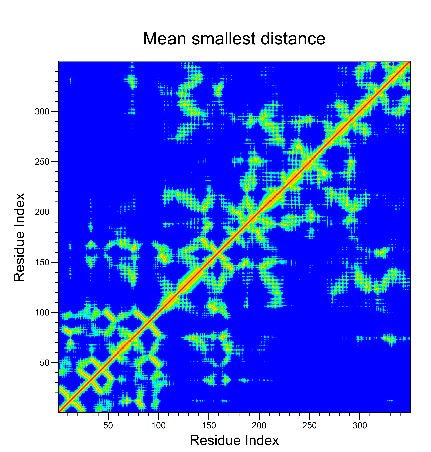 | 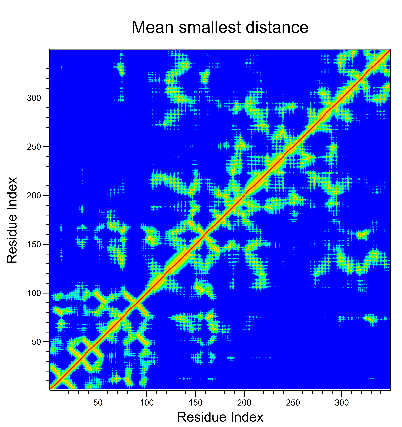 |
| E |  |  |
| 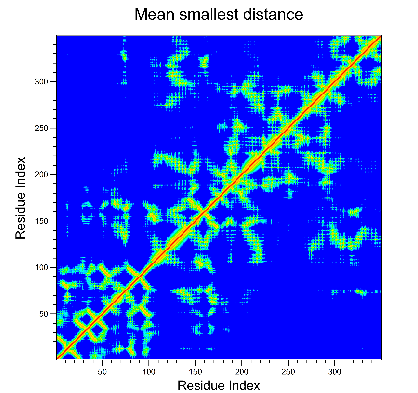 | 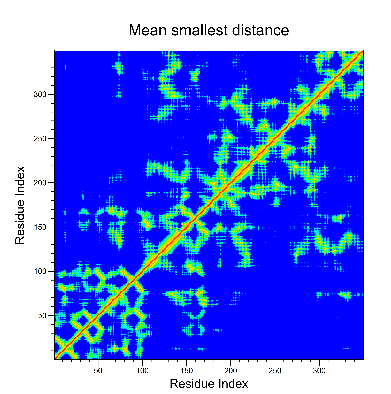 | 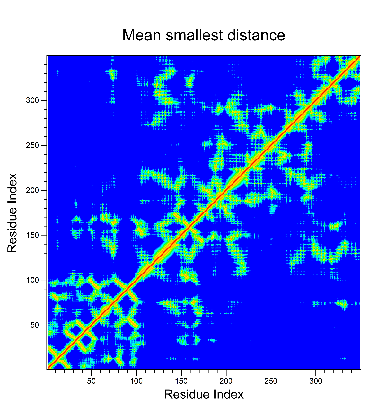 |
| F |  |  |
| 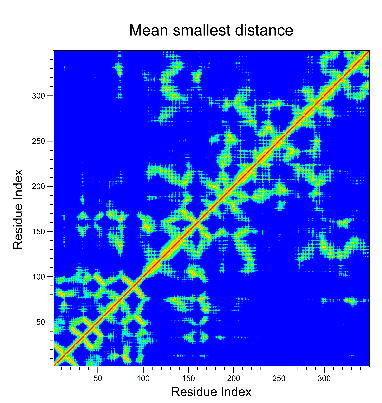 | 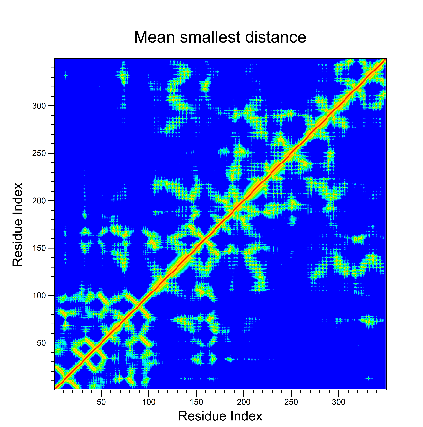 | 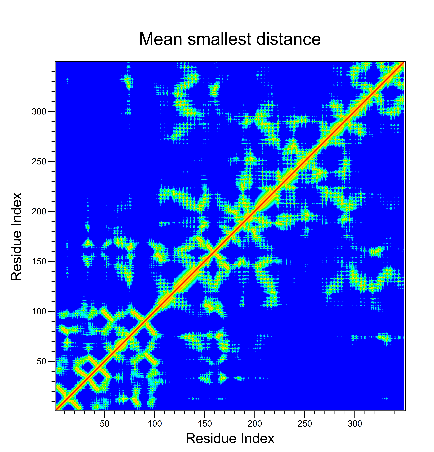 |
|  |  |  |
| Fig. S2- the conformational comparison of GSK3-β in apo (white) and in complex with ATP (A), AP(B), LO (C), RA (D), and SA (E) in three replicas. | | |
